# Ozaki technique versus Ross operation for complex aortic valve diseases in children: a retrospective cohort study

**DOI:** 10.1097/JS9.0000000000001959

**Published:** 2024-07-18

**Authors:** Guan-Xi Wang, Sen Zhang, Kai Ma, Kun-Jing Pang, Xu Wang, Ke-Ming Yang, Ben-Qing Zhang, Yang Yang, Shou-Jun Li

**Affiliations:** a Pediatric Cardiac Surgery Center, Fuwai Hospital, National Center for Cardiovascular Diseases, Chinese Academy of Medical Sciences and Peking Union Medical College, Xicheng District; b Department of Echocardiography, Fuwai Hospital, National Center for Cardiovascular Diseases, Chinese Academy of Medical Sciences and Peking Union Medical College, Xicheng District, Beijing, People’s Republic of China

**Keywords:** aortic valve regurgitation, aortic valve repair, aortic valve stenosis, OZAKI technique, Ross surgery

## Abstract

**Background:**

The Ozaki technique demonstrated promising results in adults, but few studies reported on pediatric patients with limited follow-up time. This study aimed to evaluate the mid-term results of Ozaki technique compared with Ross operation for complex aortic valve diseases in children.

**Materials and methods:**

One hundred and seventeen children underwent either Ozaki (*n*=64) or Ross (*n*=53) operation from January 2017 to December 2023. The primary endpoint was incidence of moderate or severe regurgitation/stenosis (AR/AS) post procedure.

**Results:**

No significant difference was observed in age (6.5±3.4 vs. 7.9±4.3 years) and weight (25.9±15.5 vs. 31.0±25.9 kgs) at surgery. The Ozaki group had significantly more patients in heart failure (20.3 vs. 1.9%, *P* = 0.003) before surgery and more patients needed ECMO installation (6.3% vs. 0, *P*=0.125) after surgery. The Ozaki group were in worse status with more patients occurred heart failure (20.3 vs. 1.9%, *P* = 0.003) before surgery and needed ECMO installation (6.3 vs. 0, *P* = 0.125) after surgery. During follow-up (20.4±17.3 vs. 22.7±22.8 months, *P*=0.526), five patients (7.8%) in Ozaki group but no patients in Ross group required reoperations. The incidence of moderate or severe AR (28.1 vs. 3.1%) and AS (31.3 vs. 5.7%) were significantly higher than Ross group. Multivariate analysis identified lower age [HR: 1.282 (95% CI: 1.075–1.529), *P*=0.006] and ECMO installation [HR: 0.126 (0.018–0.887), *P*=0.037] to be risk factors for moderate or severe AR, and higher aortic transvalvular gradient before discharge was confirmed as the only risk factor for moderate or severe AS (≥36 mmHg) at follow-up in Ozaki group.

**Conclusion:**

Ozaki technique may be used as a palliative procedure for complex aortic valve diseases in children, but its’ mid-term results were not durable as Ross surgery, especially younger patients.

## Introduction

Highlights

- Ozaki technique may be used as a palliative procedure for complex aortic valve disease.
- Mid-term results of Ozaki technique were not durable as Ross surgery.
- Lower age and ECMO installation were risk factors for moderate or severe AR at follow-up.
- If feasible, Ross surgery maybe the first choice for younger patients.

Currently available aortic valve (AV) substitutes were associated with suboptimal results in children, and Ross procedure was considered as the gold-standard AV replacement surgery for complex AV diseases. However, Ross surgery was limited because it placed both valves at risk of degeneration and reoperation. The reoperation rates of AV and right ventricle outflow tract were 1.60 and 1.91% per year^1^, and younger age at surgery was considered as a risk factor for reintervention^2^. In addition, children with certain conditions were not good candidates for Ross surgery. Absolute contraindications included Marfan syndrome, pulmonary valve disease, immune disorders like lupus and rheumatoid arthritis, and significant mitral valve disease. Relative contraindication (due to a higher risk of autograft dysfunction) included rheumatic valve disease, dysplastic dilated aortic root^3–5^. Besides, there was a shortage of suitable conduit for right ventricular outflow tract reconstruction in China, especially homograft, given the large number of children with congenital heart disease in the country^6^. And there was a shortage of suitable tissue or mechanical valves for children with small annulus. Hence, there was an urgent need for durable and reliable repair technique and innovative replacement solutions^1,7^.

The Ozaki procedure or named AV neo-cuspidalization, involved the complete excision of diseased native leaflets and reconstruction of the valve leaflets using autologous pericardium, offering an alternative treatment to AV replacements. It has demonstrated promising results with overall survival rate of 84.6% and freedom from reoperation rate of 95.8% at 12 years^8^. There were few reports on pediatric population with AV disease repaired by Ozaki technique^9–14^, but most of the patients were older children and the mid-term and long-term results were unknown. The technique has been applied to treat complex AV diseases in children with the intention to delay valve replacement procedure at our center. This study aimed to evaluate the mid-term results of Ozaki technique compared with Ross operation for complex AV diseases in children.

## Material and methods

### Material

This work was reported in compliance with strengthening the reporting of cohort, cross-sectional, and case–control studies in surgery (STROCSS) protocol^15^. All the children’s legal guardians have signed the informed consent for the use of personal information and medical records during hospitalization. According to the research protocol approved by the institutional review board, we conducted a retrospective review of children diagnosed with AV disease who underwent surgical procedures from January 2017 to December 2023. Inclusion criteria included patients treated with either the Ozaki technique or Ross operation. Specifically, for Ross operation patients, only those who underwent right ventricular outflow tract reconstruction using homografts were included. Patients were required to be under 18 years of age. Exclusion criteria encompassed patients who underwent AV repair, replacement, or alternative techniques, as well as those aged 18 years or older. Additionally, Ross operation patients who underwent right ventricular outflow tract reconstruction with bovine jugular vein grafts, handcrafted tri-leaflet ePTFE conduits, or other materials were excluded. Peri-operative results as well as follow-up data were recorded.

### Endpoint

The primary endpoint was AV progression including the incidence of moderate or severe regurgitation (AR), or moderate or severe stenosis (AS). Echocardiography was used to evaluate the AV function, including valve stenosis and valve regurgitation over time. Severity of AS was measured using the maximum velocities and the peak pressure gradient across the valve, and the grades were as follows: mild stenosis, peak velocity <3 m/s and peak gradient <36 mmHg; moderate stenosis, peak velocity 3–4 m/s and peak gradient 36–64 mmHg; severe stenosis, peak velocity >4 m/s and peak gradient >64 mmHg^16^. Valve regurgitation was graded as grade 0 (absent), grade 1 (trivial), grade 2 (mild), grade 3 (moderate), and grade 4 (severe) according to the features of the jet flow as measured by pulsed Doppler echocardiography^12,17^.

## Surgical technique

### The Ozaki technique

The single-leaflet^10^ and three-leaflet reconstruction^11^ utilizing Ozaki technique have been described in previous articles. All procedures were performed through full sternotomy with cardiopulmonary bypass. Autologous pericardium was carefully harvested and then meticulously attached to a stainless-steel board. The outer surface of the pericardium was thoroughly cleansed of fat and other redundant tissue. Subsequently, the excised pericardium underwent treatment with a 0.6% glutaraldehyde solution for 5–10 min, followed by three rinses with physiological saline solution, each lasting 6 min. Following heparinization, cardiopulmonary bypass was established routinely through sequential cannulations of the ascending aorta, inferior vena cava, and superior vena cava. Finally, a transverse incision through the aortic root was made to expose the AV. Inspected the pathological changes of leaflets carefully, and then determined whether to perform valve repair or leaflets reconstruction. The leaflets that were amenable for repair were meticulously excised. Different from three-leaflet or single-leaflet reconstruction technique, the number of leaflets to be reconstructed depended on the number of leaflets could not be repaired, maybe one, two, or three leaflets at our center. Then, the distance between the commissures of the damaged leaflet was measured using the sizing apparatus originally invented by Ozaki and his colleagues (JOMDD), providing appropriate tension to reproduce the annulus during diastole, upsizing rather than downsizing if in between sizes. A new leaflet of the corresponding size was trimmed from the glutaraldehyde-treated autologous pericardium using an original template. If there was no sufficient autologous pericardium, bovine pericardium patch would be another choice. Finally, the annular margin of the pericardial leaflet was sutured with a running 4-0 or 5-0 prolene sutures to the annulus. The top of the commissural coaptation was secured with additional 5-0 prolene suture, and the new junction should be slightly higher than the original junction. As a result, a new tricuspid coaptation was created. When the patient presented with a bicuspid valve deformity and symmetric development, if only one leaflet was damaged, a bicuspid valve mold may be used to create a new leaflet to replace the impaired one. Individualized surgical techniques were also used to treat the combined lesions, including ventricle septal defect repair, atrial septal defect repair, ascending aorta replacement, *et al*. After eliminating the air in the heart, removed the cross-clamp and withdrawn cardiopulmonary bypass gradually. Transesophageal echocardiography was routinely arranged to confirm the hemodynamical results in the operation room. If there was significant residual regurgitation or stenosis, reoperation would be carried out. An epicardial temporary pacemaker was placed routinely.

### Ross procedure

Ross procedure was carried out by a standard full root technique with coronary reimplantation.

### Data collection and definition

Patients’ clinical data and demographics were obtained from the electronic medical record system. Early death was defined as death occurring within 30 days after surgery. Late mortality was defined as death after 30 days or after discharge if the length of hospital stay was more than 30 days. All patients were followed up in the 1st, 3rd, 6th, and 12th months of the first year of surgery, and at least once a year thereafter.

### Statistical analysis

Categorical variables were presented as frequencies and percentages. Continuous variables were described as means with SD for normally distributed data or as medians with interquartile ranges for non-normally distributed data, assessed using the Shapiro–Wilk test. Differences in continuous variables between groups were evaluated using Student’s *t*-test for normally distributed data and the Mann–Whitney *U* test for nonparametric data. Categorical variables were compared using *χ* ^2^ statistics or Fisher’s exact test, depending on the expected cell frequencies. Survival analysis was performed using Kaplan–Meier curves, and differences between survival curves were assessed using the log-rank test. Cox proportional hazards regression analysis was employed to identify independent predictors of outcomes. All statistical analyses were conducted using SPSS version 25.0 (IBM Corp.) and GraphPad Prism software version 8.0 (GraphPad Software). Two-tailed tests of significance were applied, with statistical significance defined as *P*<0.05.

## Results

### Preoperative results

The demographics were summarized in Table 1. A total of 117 patients were included in the study: 64 patients in Ozaki group and 53 patients in Ross group. No significant difference was observed in average age (6.5±3.4 vs. 7.9±4.3 years, *P*=0.062) and weight (25.9±15.5 vs. 31.0±25.9 kgs, *P*=0.187) at surgery between the two groups. A total of 44 patients received at least one surgery previously.

**Table 1:** Demographic characteristics of all the patients.

Variables	Ozaki (*n*=64)	Ross (*n*=53)	*P*
Male (*n*, %)	44 (68.8%)	35 (66.0%)	0.843
Age (years)	6.5±3.4	7.9±4.3	0.062
Weight (Kg)	25.9±15.5	31.0±25.9	0.187
Preoperative aortic valve peak velocity (m/s)	2.5±1.3	4.7±1.2	0.000
Preoperative aortic valve annular diameter (mm)	17.4±3.4	16.9±3.2	0.356
Previous operations	26 (40.6%)	18 (34.0%)	0.566
Aortic valve repair (*n*, %)	10 (15.6%)	7 (13.2%)	0.796
VSD repair (*n*, %)	10 (15.6%)	1 (1.9%)	0.012
VSD occlusion (*n*, %)	3 (4.7%)	0	0.450
Excision of discrete subaortic membrane (*n*, %)	3 (4.7%)	2 (3.8%)	1.000
PDA ligation (*n*, %)	3 (4.7%)	2 (3.8%)	1.000
Mitral valve repair (*n*, %)	2 (3.1%)	2 (3.8%)	1.000
Tricuspid valve repair (*n*, %)	2 (3.1%)	0	0.5000
DORV repair (*n*, %)	1 (1.6%)	0	1.000
Abnormal muscle resection of RVOT (*n*, %)	1 (1.6%)	0	1.000
David operation (*n*, %)	1 (1.6%)	0	1.000
Supravalvular aortic stenosis repair (*n*, %)	1 (1.6%)	4 (7.5%)	0.174
Patent foramen ovale repair (*n*, %)	1 (1.6%)	2 (3.8%)	0.589
Ascending aortoplasty (*n*, %)	1 (1.6%)	1 (1.9%)	1.000
pulmonary arterioplasty (*n*, %)	1 (1.6%)	1 (1.9%)	1.000
Abnormal muscle resection of LVOT (*n*, %)	1 (1.6%)	2 (3.8%)	0.589
ASD repair (*n*, %)	1 (1.6%)	0	1.000
Aortic balloon valvuloplasty (*n*, %)	0	5 (9.4%)	0.017
Permanent pacemaker implantation (*n*, %)	0	1 (1.9%)	0.453
Aortic valve replacement (*n*, %)	0	1 (1.9%)	0.453
Main causes for this operation			
Congenital valvular deformity (*n*, %)	36 (56.3%)	51 (96.2%)	0.000
Surgical injury (*n*, %)	15 (23.4%)	1 (1.9%)	0.001
Infectious endocarditis (*n*, %)	10 (15.6%)	1 (1.9%)	0.012
Marfan’s syndrome (*n*, %)	3 (4.7%)	0	0.250
Rheumatic heart disease (*n*, %)	1 (1.6%)	0	1.000
Behcet’s disease (*n*, %)	1 (1.6%)	0	1.000
Pathological manifestation			
Combined with moderate or severe AR (*n*, %)	53 (82.8%)	15 (28.3%)	0.000
Combined with moderate or severe AS (*n*, %)	19 (29.7%)	50 (94.3%)	0.000
Preoperative status			
Invasive mechanical ventilation (*n*, %)	2 (3.1%)	0	0.500
Cardiac insufficiency (*n*, %)	13 (20.3%)	1 (1.9%)	0.003

* ASD, atrial septal defect; DORV, double outlet of right ventricle; LVOT, left ventricle outflow tract; PDA, patent ductus arteriosus; RVOT, right ventricle outflow tract; VSD, ventricular septal defect.

There was significant difference in the distribution of main causes for this operation. The main causes in Ozaki group were congenital valvular deformity (56.3%), surgical injury (23.4%), infectious endocarditis (15.6%), Marfan’s syndrome (4.7%), rheumatic heart disease (1.6%), Behcet’s disease (1.6%), while the main causes in Ross group were congenital valvular deformity (96.2%), surgical injury (1.9%), and infectious endocarditis (1.9%). Moderate or severe AR demonstrated in 53 (82.8%) patients in Ozaki group and 15 (28.3%) patients in Ross group, *P*<0.001. While moderate or severe AS demonstrated in 19 (29.7%) patients in Ozaki group and 50 (94.3%) patients in Ross group, *P*<0.001.

Most of the patients were in stable state, but 13 (20.3%) patients presented with heart failure (LVEF <50%) and two (4.0%) patients received endotracheal intubation before surgery in Ozaki group. Only one patient (1.9%) presented with heart failure and no patient received endotracheal intubation before surgery in Ross group.

### Intraoperative results

The demographics were summarized in Table 2. The annulus, leaflets, and sub-valvar apparatus were evaluated carefully to determine the precise anatomy of the lesions. There was significant difference in the distribution of major anatomical pathologies, including mono-cuspid valve (1.6%), bicuspid valve (26.6%), tricuspid valve (67.2%), and quadricuspid valve (4.7%) in Ozaki group, while mono-cuspid valve (1.9%), bicuspid valve (86.8%), tricuspid valve (9.4%), and quadricuspid valve (1.9%) in Ross group, *P*<0.001.

**Table 2:** Inoperative and postoperative characteristics.

Variables	Ozaki (*n*=64)	Ross (*n*=53)	*P*
Aortic valve anatomy			0.000
Monocuspid (*n*, %)	1 (1.6%)	1 (1.9%)	1.000
Bicuspid (*n*, %)	17 (26.6%)	46 (86.8%)	0.000
Tricuspid (*n*, %)	43 (67.2%)	5 (9.4%)	0.000
Quadricuspid (*n*, %)	3 (4.7%)	1 (1.9%)	0.625
Number of replaced leaflets			
Single leaflet reconstruction (*n*, %)	36 (56.3%)		
Two leaflets reconstruction (*n*, %)	17 (26.6%)		
Three leaflets reconstruction (*n*, %)	11 (17.2%)		
Left coronary cusp reconstruction (*n*, %)	26 (40.6%)		
Right coronary cusp reconstruction (*n*, %)	43 (67.2%)		
Noncoronary cusp reconstruction (*n*, %)	37 (53.1%)		
New leaflet material			
Autologous pericardium (*n*, %)	58(90.6%)		
Bovine pericardium (*n*, %)	6(9.4%)		
Valve coaptation			
Two leaflets coaptation (*n*, %)	2 (3.1%)		
Three leaflets coaptation (*n*, %)	62 (96.9%)		
Cardiopulmonary bypass time (minutes)	152.7±74.5	191.7±48.8	0.001
Aortic cross-clamp time (minutes)	97.4±47.6	135.1±36.1	0.000
Concomitant procedures	38 (59.4%)	13 (24.5%)	0.000
VSD repair (*n*, %)	9 (14.1%)	0	0.004
Supravalvular aortic stenosis repair (*n*, %)	8 (12.5%)	2 (3.8%)	0.110
Patent foramen ovale repair (*n*, %)	7 (10.9%)	2 (3.8%)	0.180
Excision of discrete subaortic membrane (*n*, %)	6 (9.4%)	3 (5.7%)	0.509
Tricuspid valve repair (*n*, %)	6 (9.4%)	1 (1.9%)	0.125
Mitral valve repair (*n*, %)	5 (7.8%)	2 (3.8%)	0.454
Ascending aortoplasty (*n*, %)	4 (6.3%)	2 (3.8%)	0.688
ECMO installation (*n*, %)	4 (6.3%)	0	0.125
Abnormal muscle resection of LVOT (*n*, %)	4(6.3%)	2(3.8%)	0.688
Modified Konno operation (*n*, %)	3(4.7%)	1(1.9%)	0.625
Removal of VSD occluder (*n*, %)	3(4.7%)	0	0.250
Coronary artery opening plasty (*n*, %)	2(3.1%)	0	0.500
PDA ligation (*n*, %)	2(3.1%)	1(1.9%)	1.000
Prosthetic vessel replacement (*n*, %)	2(3.1%)	0	0.500
Mitral valve suprapvalvular membrane excision	1 (1.6%)	1 (1.9%)	1.000
Abnormal muscle resection of RVOT (*n*, %)	1 (1.6%)	0	1.000
ASD repair (*n*, %)	1 (1.6%)	0	1.000
Aortic coarctation repair (*n*, %)	1 (1.6%)	1 (1.9%)	1.000
Modified Morrow operation (*n*, %)	0	1 (1.9%)	0.453
Mechanical ventilation time (hours)	62.3±140.4	32.7±100.2	0.188
ICU time (days)	5.1±7.9	4.1±5.8	0.426

The number of leaflets that could not be repaired during the surgery determined the number of leaflets that needed to be reconstructed. The distribution was as follows: single leaflet reconstruction in 36 (56.3%) patients, two leaflets reconstruction in 17 (26.6%) patients, and three leaflets reconstruction in 11 (17.2%) patients. Among them, 26 patients (40.6%) had the left coronary cusp reconstructed, 43 patients (67.2%) had the right coronary cusp reconstructed, and 34 patients (53.1%) had the noncoronary cusp reconstructed. Glutaraldehyde-treated autologous pericardium was adopted to create leaflets in most of (90.6%) the patients. Sometimes there was a shortage of autologous pericardium after previous surgeries, and bovine pericardium patch was used as an alternative in six (9.4%) patients. As a result, the AV presented as three-leaflet coaptation in 62 patients, and presented as two-leaflet coaptation in two patients.

Fifty-one patients performed concomitant procedures at the time of AV surgery in all the cohorts. Because the Ozaki procedure was relatively easier to perform, cardiopulmonary bypass time (152.7±74.5 vs. 191.7±48.8 min, *P*=0.001) and aortic cross-clamp time (97.4±47.6 vs. 135.1±36.1, *P*<0.001) were shorter compared with Ross group. Four patients required ECMO installation for severe heart failure after heart resuscitating in Ozaki group. The mechanical ventilation time was 62.3±140.4 vs. 32.7±100.2 h, *P*=0.188; and postoperative ICU stay time was 5.1±7.9 vs. 4.1±5.8 days, *P*=0.426.

### Early and late death

Three patients (4.7%) experienced early death due to heart failure after surgery in the Ozaki group, whereas no patients experienced early death in the Ross group (*P*=0.250).

**Figure 1:**
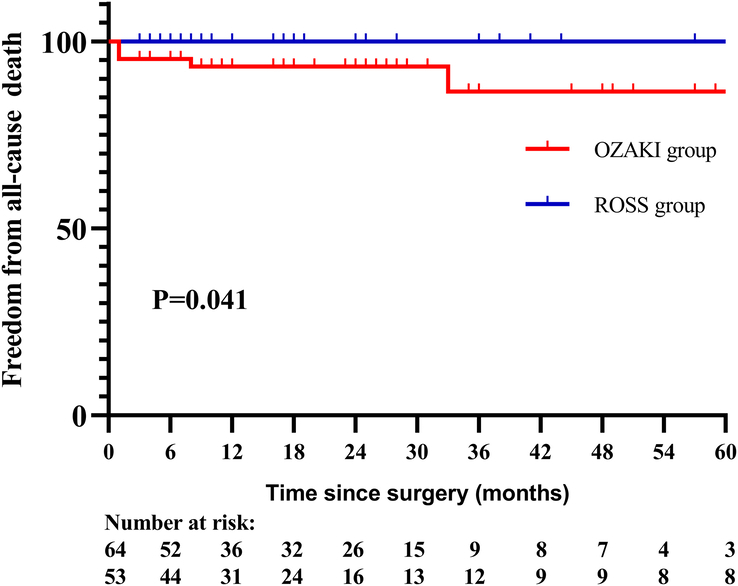
Freedom from death between the two groups.

During the follow-up period (20.4±17.3 vs. 22.7±22.8 months, *P*=0.526), two patients experienced late death in the Ozaki group. One patient died due to a pulmonary hypertensive crisis 8 months after discharge, and another patient experienced sudden death for unknown reasons 33 months after discharge. No patients experienced late death in the Ross group. The estimated 1-year, 3-year, and 5-year survival rates were 93.2, 86.6, and 86.6%, respectively, in the Ozaki group, and 100.0, 100.0, and 100.0% in the Ross group, respectively (log-rank *P*=0.041, Fig. 1).

### Reoperation

**Figure 2:**
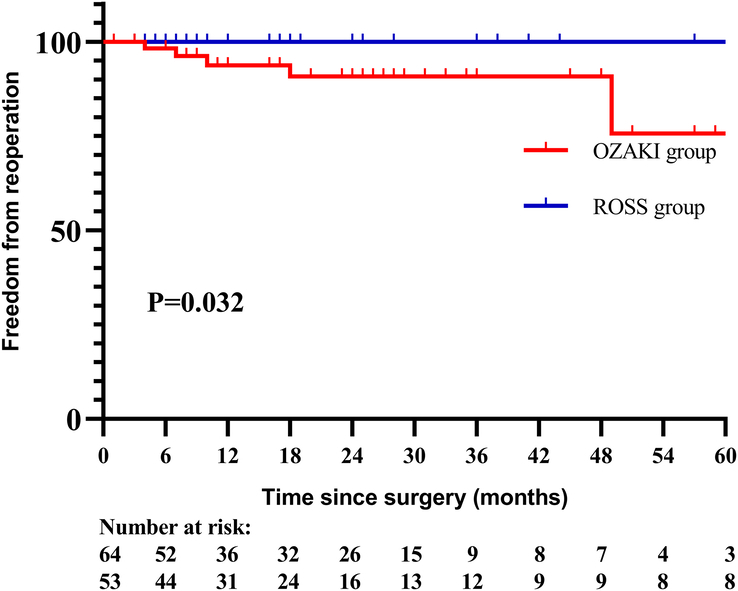
Freedom from reoperation between the two groups.

Five patients in Ozaki group, but no patient in Ross group needed reoperations. Two patients underwent Ozaki surgery due to endocarditis 4 and 7 months after previous surgery. Three patients demonstrated severe AR due to structural valve degeneration with decreased mobility, and developed clinical signs of heart failure. They received mechanical valve replacements 10, 18, 49 months after previous surgery. It was estimated freedom from reoperation rate at 1-year, 3-year, 5-year was 93.7, 90.8, 75.7% in Ozaki group, and 100.0, 100.0, 100.0% in Ross group respectively, log rank *P*=0.032 (Fig. 2).

## Aortic valve function

### Aortic valve stenosis

**Figure 3:**
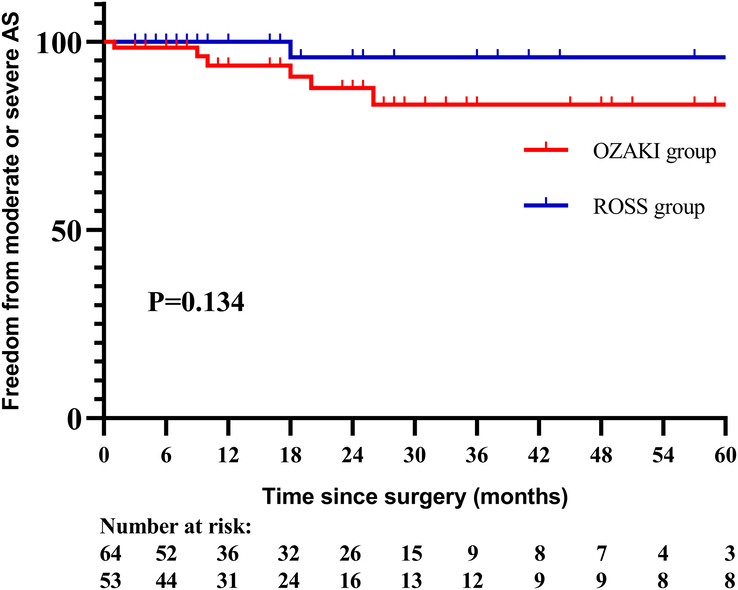
Freedom from moderate or severe AS between the two groups.

There were six (9.4%) patients in Ozaki group, and only one patient (1.9%) in Ross group demonstrated moderate or severe aortic AS, *P*=0.125. A Cox regression model was used to analyze the risk factors for moderate or severe AS in Ozaki group, and univariate analysis identified higher peak transvalvular aortic velocity before discharge was the only risk factor for moderate or severe AS at follow-up, [HR: 8.958; 95% CI (2.235–35.900), *P*=0.002]. It was estimated freedom from moderate or severe AS at 1, 3, 5 years were 93.6%, 83.3%; 83.3% in Ozaki group, and 100.0%, 95.8%, 95.8% in Ross group, log rank *P*=0.134 (Fig. 3).

### Aortic valve regurgitation

There were 18 (28.1%) patients in Ozaki group, and two (3.8%) patients in Ross group demonstrated moderate or severe AR during follow-up. The main causes for moderate or severe AR in Ozaki group were as follows: infectious endocarditis in two patients, structural valve degeneration (decreased mobility) in 15 patients, and severe aortic dilation in one patient. The patient with severe aortic dilation was a 6-year-old boy, and he was diagnosed as rheumatic heart disease in the acute phase. He was in unstable state with severe heart failure, and received emergency surgery. The AV annulus and aortic wall were unstable after three leaflets reconstruction, and the AR increased rapidly from little to severe. Unfortunately, the patient recovered badly and died for congestive heart failure before discharge. It was not a technical failure of leaflet reconstruction. The main causes for moderate or severe AR in Ross group were neo-aortic root dilatation.

**Figure 4:**
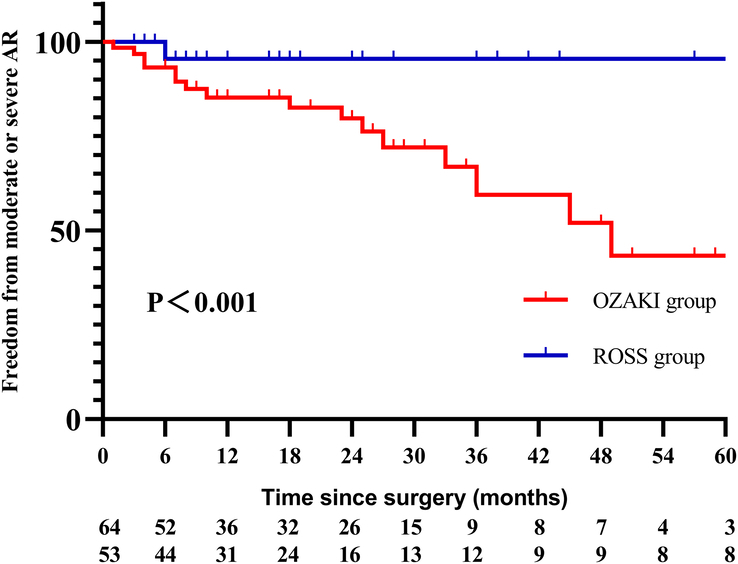
Freedom from moderate or severe AR between the two groups.

A Cox regression model was used to analyze the risk factors for moderate or severe AR in Ozaki group. Univariate analysis of variables allowed the selection of variables (*P*<0.10) for inclusion in the multivariate analysis. Multivariate analysis identified younger age at surgery [1.282 (1.075–1.529), *P*=0.006] and ECMO installation [0.126 (0.018–0.887), *P*=0.037] to be risk factors for moderate or severe AR at follow-up (Table 3). It was estimated freedom from moderate or severe AR at 1, 3, 5 years were 83.2%, 60.0%; 43.7% in Ozaki group, and 95.5%, 91.5%, 91.5% in Ross group, log rank *P*<0.001 (Fig. 4).

**Table 3:** Risk analysis for moderate or severe AR after surgery in Ozaki group (Cox regression).

	Univariate		Multivariate	
Variables	HR (95% CI)	*P*	HR (95% CI)	*P*
Age at surgery (months)	**1.171** (**1.009–1.359)**	**0.038**	**1.282** (**1.075–1.529)**	**0.006**
Weight at surgery (kg)	1.103 (0.986–1.040)	0.348		
Have at least one surgery previously	0.921 (0.317–2.678)	0.879		
Preoperative LV ejection fraction <50%	0.453 (0.138–1.484)	0.191		
Main causes for this operation				
(congenital valvular deformity)	**0.329** (**0.107–1.013)**	**0.053**	0.374 (0.096–1.458)	0.157
Combined with moderate or severe AS	**0.312** (**0.103–0.940)**	**0.039**	1.528 (0.313–7.475)	0.600
Combined with moderate or severe AR	2.306 (0.750–7.098)	0.145		
ECMO installation	**0.217** (**0.058–0.807)**	**0.023**	**0.126** (**0.018–0.887)**	**0.037**
Aortic valve anatomy (Bicuspid valve)	**0.169** (**0.051–0.558)**	**0.004**	0.282 (0.024–3.323)	0.314
Aortic valve anatomy (Tricuspid valve)	**3.293** (**1.188–9.130)**	**0.022**	0.877 (0.097–7.922)	0.907
Number of replaced leaflets	**1.211** (**0.671–2.186)**	**0.525**		
New leaflet material (autologous pericardium)	**3.470** (**0.936–12.872)**	**0.063**	2.006 (0.395–10.175)	0.401
Preoperative AV annular diameter (mm)	1.017 (0.883–1.172)	0.816		

## Pulmonary valve function

### Pulmonary valve stenosis

No patient occurred moderate or severe pulmonary valve stenosis in both groups.

### Pulmonary valve regurgitation

No patients in Ozaki group, but eight (15.1%) patients in Ross group occurred moderate or severe pulmonary valve regurgitation during follow-up. It was estimated freedom from moderate or severe pulmonary regurgitation at 1, 3, 5 years were 100.0%, 79.1%; 70.3% in Ross group, significantly inferior to patients in Ozaki group, log rank *P*=0.011.

### Comparison after excluding patients with preoperative cardiac insufficiency

Considering disparities in the preoperative cardiac status between the two groups could potentially bias the postoperative outcomes, we excluded patients with preoperative cardiac insufficiency from both groups and conducted a recomparison. A total of 101 patients were included: 51 patients in Ozaki group and 52 patients in Ross group. Both groups had no early death, late death, and reoperations.

**Figure 5:**
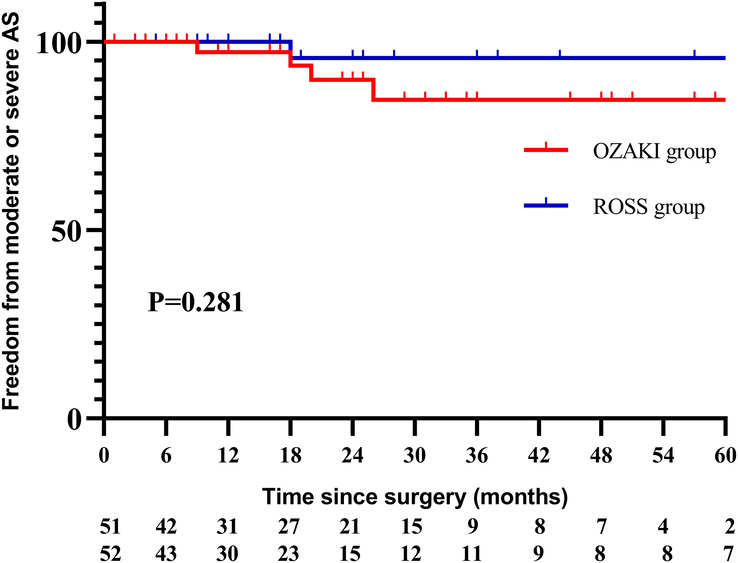
Freedom from moderate or severe AS between the two groups (excluding patients with preoperative cardiac insufficiency).

**Figure 6:**
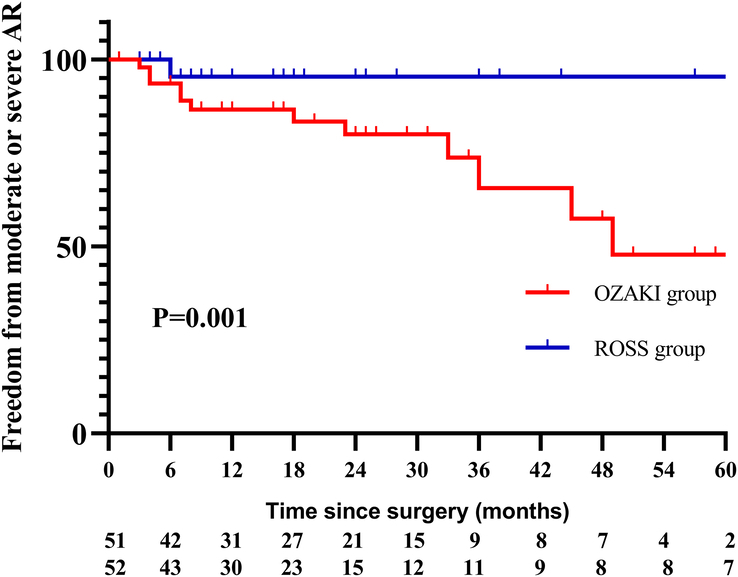
Freedom from moderate or severe AR between the two groups (excluding patients with preoperative cardiac insufficiency).

During the follow-up period (20.0±18.1 vs. 22.4±22.9 months, *P*=0.921), there were four (7.8%) patients in Ozaki group, and only one patient (1.9%) in Ross group demonstrated moderate or severe aortic AS, *P*=0.205. It was estimated freedom from moderate or severe AS at 1, 3, 5 years were 97.2%, 84.6%; 84.6% in Ozaki group, and 100.0%, 95.5%, 95.7% in Ross group, log rank *P*=0.281 (Fig. 5). There were 14 (27.5%) patients in Ozaki group, and two (3.8%) patients in Ross group demonstrated moderate or severe AR, *P*=0.001. It was estimated freedom from moderate or severe AR at 1, 3, 5 years were 86.6%, 65.6%; 47.8% in Ozaki group, and 95.3%, 95.3%, 95.3% in Ross group, log rank *P*=0.001 (Fig. 6).

## Discussion

Our center tended to prefer AV reconstruction over mechanical valve replacement for several reasons. The main concern was there was a shortage of suitable mechanical valves for small annulus and it required long-term administration of anticoagulant medication such as warfarin. Besides, managing anticoagulant medication could be challenging for young children, increasing the risk of complications. And most parents were hesitant to choose mechanical valve replacement and they preferred to wait until their child was older and capable of self-managing anticoagulant medication. In this scenario, the Ozaki technique provided a new alternative for addressing these complex conditions.

As a new method of AV repair, Ozaki technique expanded surgical indications for complex AV diseases, and it has been applied as a promising alternative solution in the pediatric population^9–14^. Compared with conventional mechanical or bioprosthetic valves, it had the advantages of preserving the natural motion of the AV anulus and the coordination of the left ventricle, sinus of Valsalva and the aorta, hence, it had the natural aortic root expansion in the systole with maximal effective orifice area^18,19^. Compared with Ross procedure, the Ozaki procedure restored a physiological laminar flow pattern and showed similar hemodynamic results in the short-term period^20^.

According to the available literature about Ozaki technique, our cohort had the largest sample size in children, the youngest average age (7.9±4.3 years, range: 0.9–15.0 years) at surgery, the worst status before surgery, and the longest follow-up after surgery. Almost half of the patients have received at least once surgery previously, and they were faced with high risk of thoracotomy. Besides congenital valvular deformity, surgical injury and infectious endocarditis, Ozaki technique was used to treat five complex cases who were so young to have suitable mechanical valve replacement, including three cases of Marfan’s syndrome, one case of rheumatic heart disease, one case of Behcet’s disease. Thirteen patients occurred heart failure and two patients received endotracheal intubation before surgery, and four patients needed ECMO installation in Ozaki group. All of them belonged to absolute or relative contraindication of Ross procedure^3–5^. Overall, the patients in Ozaki group had complex conditions, worse preoperative status, and higher risk of death compared with Ross surgery. The Ozaki procedure was relatively simple with the advantages of shorter cardiopulmonary bypass time and aortic cross-clamp time, and it could be used as a palliative procedure for complex AV diseases to delay AV replacement.

Boston Children’s Hospital first applied the technique to pediatric patients who were unable to undergo the Ross procedure due to conditions such as truncus arteriosus, aortic regurgitation, or a severely dilated aortic annulus. Baird *et al*. reported their three-leaflet reconstruction experience. Among the 57 patients, the median age was 12.43 (range: 0.7–25.4) years and 48 cases were under 18 years old. At median follow-up of 8.1 months, 96% and 91% of patients had less than moderate regurgitation and stenosis, respectively^11^. While the original technique involved all the three aortic leaflets, Marathe *et al*. began to undergo the single AV leaflet reconstruction attempting to retain native leaflet tissue, with several advantages including maintaining single valve disease, somatic growth potential, preserved dynamic annular motion and limited need for long-term anticoagulation^10^. Among the 33 patients, the median age was 9.3 (range: 1.1–25.4) years. Freedom from more than moderate AR and AS at 2 years was 76 and 86% with a median follow-up of 1.1 years. Wiggins *et al*. from the Great Ormond Street Hospital reported 58 patients with a median age of 14.8 (interquartile range: 10.6–16.8) years that underwent three leaflets and single leaflet reconstruction. Freedom from reoperation or moderate and greater AR was 79% at 3 years^12^. Most of the patients mentioned above were older children (average annular diameter of 20 mm). Although the follow-up time has been so short (1–2 years), many patients were still at risk of stenosis or regurgitation.

With our medium-term results, the Ozaki technique was not durable as Ross surgery, especially younger patients. It presented some special challenges in children under 6 years of age, including the risk of coronary ostial obstruction from redundant leaflets, valve stenosis from bulky leaflets relative to aortic root and changes in leaflet material with stiffness, retraction^21^. Hence, Ross surgery may be advised firstly for younger patients if possible. Besides lower age, extracorporeal membrane oxygenation installation at surgery was identified as risk factors for moderate or severe regurgitation at follow-up. It may be related to the adverse impact of blood flow from aortic cannulation on the new leaflets. Contrary to our results, a study about the older children with a median age 12.4 (8.8–15.8) years. It showed Ozaki technique could be used as an alternative solution for Ross surgery, and there was no significant difference in freedom from reoperation or death between the Ozaki technique and Ross procedure^22^. The different results may contribute to the older age and the shorter follow-up time in Ozaki group, but longer follow-up time [38.9 (13.8–52.8) months vs. 11.3 (4.7–21) months, *P*=0.02] in Ross group. Longer follow-up was needed.

Peak AV gradient at follow-up was significantly related to preoperative aortic annular size^23^. For patients with small annulus, annular enlargement procedure was always needed^11,14^. Konno operation was adopted and patched with pericardium in three patients, and the early and mid-term results were satisfactory. It should be noted the leaflet size must be precisely determined, and the sinus and sinotubular junction dimensions cannot be undersized. Glutaraldehyde treated autologous pericardium was the first choice to make new leaflets, and the use of heterologous pericardium should probably be minimized^23,24^. The use of bovine pericardium was significantly associated with higher peak AV gradient^23^. But bovine pericardium could also contribute to similar results compared with autologous pericardium^12^. Longer term follow-up was required to ascertain definitive results.

Besides structural valve degeneration of the valves, infective endocarditis was one of the main reasons for reoperation^16^. Wiggins *et al*. reported there were six late reoperations, and 50% were due to endocarditis. Of the three patients, one patient had reoperation following 6 weeks of antibiotic treatment with closure of peri-annular abscess cavities, and one patient received a mechanical valve implantation, and one had a homograft root replacement for associated aortic root destruction. In our cohort, two patients received OZAKI procedure again. Redo OZAKI technique could be great advantages for young patients who were highly prone to recurrence of infection, because the leaflets can be easily removed and there was no sewing cuff that could be exposed to blood-dissolved antibiotics^25^. Acceptable mid-term prognosis was observed, but further follow-up was needed.

### Limitations

This retrospective real-world study had several limitations. Firstly, the Ozaki group exhibited a higher incidence of preoperative heart failure (20.3 vs. 1.9%, *P*=0.003), and a greater postoperative requirement for ECMO (6.3 vs. 0, *P*=0.125). These differences indicated disparities in preoperative cardiac status between the two groups, potentially impacting postoperative outcomes. The higher proportion of critically ill patients in the Ozaki group may be associated with the simplicity of the technique, with the advantages of shorter cardiopulmonary bypass and aortic cross-clamp times. Consequently, it was used as a palliative procedure to delay AV replacement for complex AV diseases.

To enhance the credibility of our research, multicenter studies to expand the sample size and propensity-matched study methods to reduce bias were necessary. Additionally, further investigation into the long-term effects of surgery was warranted.

## Conclusion

Ozaki technique may be used as a palliative procedure for complex AV diseases in children, but its’ mid-term results was not durable as Ross surgery, especially younger patients. It should be carefully selected for children, if feasible, Ross surgery maybe the first choice for complex AV diseases.

## Ethical approval

Patients were retrieved from the Chinese Database for Congenital Heart Surgery database (Registry No. ChiCTR1800014577). Ethical approval for this study (ethical number: 2017–977) was provided by the Ethical Committee of Fuwai Hospital, Beijing, China on 30 November 2019.

## Consent

Written informed consent was obtained from the patient's parents/legal guardian for publication and any accompanying images. A copy of the written consent is available for review by the Editor-in-Chief of this journal on request.

## Source of funding

This study was supported by National Key R & D Program of China (No. 2017YFC1308100); National Clinical Research Center for Cardiovascular Diseases, Fuwai Hospital, Chinese Academy of Medical Sciences (No. NCRC2024001); the Youth Science Foundation of Fuwai hospital, Chinese Academy of Medical Sciences (No. 2022-FWQN12), and the National High Level Hospital Clinical Research Funding (No. 2023-GSP-GG-19).

## Author contribution

Contributing to the conception and design: G.-X.W., S.-J.Z. and S.L. Data collection, analysis, and interpretation: G.-X.W., S.Z., K.M., K.-J.P., X.W., K.-M.Y., B.Z., Y.Y. Writing the original draft: G.-X.W. Revising the article: S.Z. and S.L. Supervision: S.L. Validation: S.-J.L. Funding acquisition:, S.-J.L., S.Z., B.-Q.Z. Project administration: S.-J.L.

## Conflicts of interest disclosure

The authors declare that they have no financial conflict of interest with regard to the content of this report.

## Research registration unique identifying number (UIN)

Not applicable.

## Guarantor

Shoujun Li.

## Data availability statement

Any datasets generated or analyzed during the current study are publicly available upon reasonable request.

## Provenance and peer review

Not commissioned, externally peer-reviewed.
